# The efficacy and safety of tislelizumab in the treatment of locally advanced or metastatic lung cancer: a systematic review and meta-analysis

**DOI:** 10.3389/fphar.2025.1671018

**Published:** 2025-10-28

**Authors:** Yue Zhou, Sanmao Liu, Shaochu Zheng, Jiahui Han, Haizhu Huang, Jinliang Kong

**Affiliations:** ^1^ Department of Respiratory Medicine, The First Affiliated Hospital of Guangxi Medical University, Nanning, Guangxi Zhuang, China; ^2^ Division of Spinal Surgery, The First Affiliated Hospital of Guangxi Medical University, Nanning, Guangxi Zhuang, China

**Keywords:** tislelizumab, LC, efficacy, safety, meta-analysis

## Abstract

**Objective:**

The clinical role of Tislelizumab in patients with locally advanced or metastatic lung cancer (LC) remains controversial. This study aims to systematically evaluate the efficacy and safety of Tislelizumab in treating these patients through a meta-analysis.

**Methods:**

Databases including PubMed, Cochrane Library, Embase, and Web of Science were searched up to 19 May 2025. Randomized controlled trials (RCTs) and single-arm studies assessing the efficacy and safety of Tislelizumab for locally advanced or metastatic LC were included. Literature screening and data extraction were performed according to the PRISMA guidelines, and pooled odds ratios (OR) and their 95% confidence intervals (CI) were calculated using STATA 15.0 software.

**Results:**

A total of 8 studies were included, of which 5 were RCTs and 3 were single-arm studies. In the RCT subgroup, the Tislelizumab group demonstrated a higher objective response rate (ORR) [OR = 2.29, 95%CI(1.43,3.64), P = 0.001] and disease control rate (DCR) [OR = 1.64, 95%CI(1.30,2.07), P < 0.001] compared to the control group, but no significant differences were found in overall survival (OS) [OR = 0.81, 95%CI(0.60,1.10), P = 0.179] or progression-free survival (PFS) [OR = 0.74, 95%CI(0.39,1.41), P = 0.364]. Single-arm study data indicated that Tislelizumab treatment achieved a high ORR [OR = 0.54, 95%CI (0.34,0.74), P < 0.001] and DCR [OR = 0.86, 95%CI (0.78,0.92), P < 0.001]. Subgroup analysis revealed that Tislelizumab had similar effects on ORR and DCR in non-small cell lung cancer (NSCLC) and small cell lung cancer (SCLC).

**Conclusion:**

The meta-analysis results suggest that Tislelizumab demonstrates significant short-term efficacy (ORR and DCR) in patients with locally advanced or metastatic LC. However, the existing evidence is inadequate to confirm its long-term survival benefits (OS and PFS), and more high-quality studies are needed for validation.

## 1 Introduction

LC is one of the most prevalent malignant tumors globally, characterized by the highest incidence and mortality rates. Among these, NSCLC and SCLC are the most common pathological types (Abu Rous et al., 2023; Allemani et al., 2018; Frödin, 1996; Sullivan, 2018). For patients with locally advanced or metastatic LC, despite continuous advancements in treatment modalities, the prognosis remains poor, highlighting the urgent need for more effective therapeutic strategies. Currently, clinical management primarily relies on comprehensive approaches, including chemotherapy (Li et al., 2023; Legha et al., 1977; Nagasaka and Gadgeel, 2018), radiotherapy (Vinod and Hau, 2020; Schild, 2020), brachytherapy (International BR, 2023; Lin et al., 2023), and targeted therapy (Herrera-Juárez et al., 2023; Wu and Lin, 2022; Meyer et al., 2024). In standard treatment protocols, chemotherapy remains a cornerstone, particularly for patients without driver gene mutations or those with SCLC. However, traditional chemotherapeutic agents often cause significant systemic toxicities, such as myelosuppression, nausea, vomiting, and alopecia, with limited efficacy leading to drug resistance and disease progression (Sha et al., 2015; Wang et al., 2019a). Radiotherapy plays a pivotal role in the radical or palliative treatment of locally advanced NSCLC, but it also carries the risk of local recurrence and may cause complications like radiation pneumonitis and esophagitis (Liu et al., 2023; Mutsaers et al., 2023). With advancements in molecular biology research, targeted therapy drugs against specific driver genes (e.g., EGFR, ALK) have significantly improved survival in corresponding subtypes of patients (Garassino et al., 2023; de Castro et al., 2023). However, these drugs often face acquired resistance and are only applicable to a small proportion of patients carrying specific gene mutations (Fu et al., 2022; Xiang et al., 2024).

**FIGURE 1:**
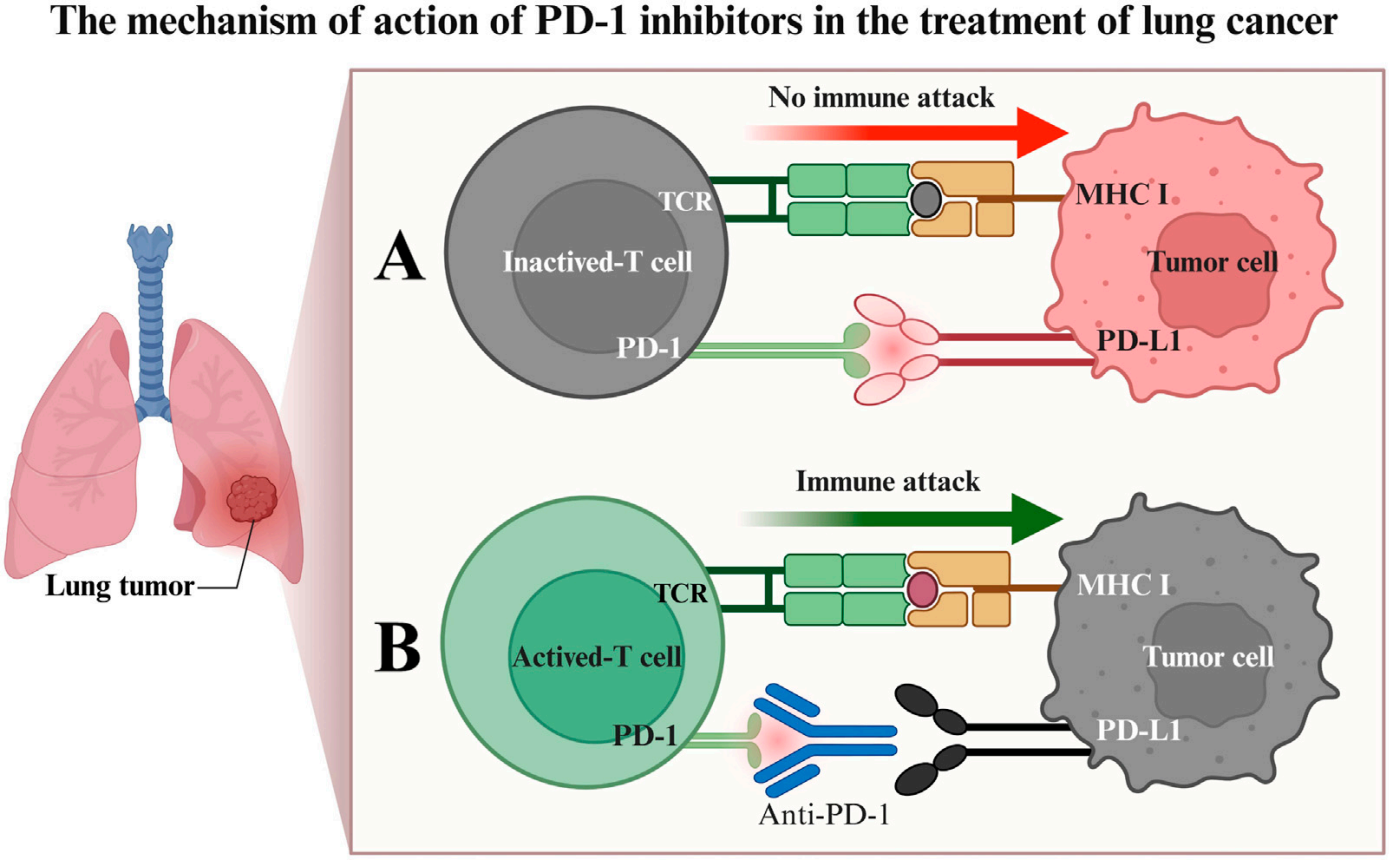
Mechanism of PD-1 inhibitor therapy for lung cancer. **(A)** Binding of the PD-1 receptor to the PD-L1 ligand results in T-cell inactivation, impairing the ability of T cells to attack tumor cells immunologically; **(B)** PD-1 inhibitors competitively bind to the PD-1 receptor, blocking its interaction with PD-L1. This preserves T-cell activity, enabling T cells to initiate an immune attack against tumor cells, leading to tumor cell apoptosis.

In recent years, the advent of immune checkpoint inhibitors (ICIs) has revolutionized LC treatment (Tang et al., 2022; Passaro et al., 2022; Konen et al., 2024), particularly antibodies targeting programmed death receptor 1 (PD-1) or its ligand (PD-L1), which have significantly improved survival outcomes in some patients (Yin et al., 2022; Chen et al., 2024; Cho et al., 2020). PD-1 is an immunosuppressive receptor expressed on activated T lymphocytes. In the tumor microenvironment, tumor cells or immunosuppressive cells highly express PD-L1, which, upon binding to PD-1 on T cells, transmits inhibitory signals, leading to T-cell exhaustion and impaired proliferation, thereby facilitating tumor evasion from immune recognition and clearance (Cheng W. et al., 2024; Cheng et al., 2022; Figure 1). Tislelizumab is a humanized IgG4 anti-PD-1 monoclonal antibody, uniquely designed to block the binding of PD-1 to PD-L1/PD-L2 with high specificity, thereby alleviating the suppression of T cells by the PD-1 pathway. More specifically, Tislelizumab has low affinity for Fc receptors (FcRs) on macrophages via its Fc segment, which reduces the potential interference of antibody-dependent cell-mediated phagocytosis (ADCP) on pharmacodynamic effects, theoretically aiding in more effectively maintaining T-cell function. By restoring T-lymphocyte-mediated immune responses, Tislelizumab aims to reactivate anti-tumor immune responses (Zhao et al., 2024; Daei Sorkhabi et al., 2023; Cheng Y. et al., 2024).

Based on this mechanism of action, multiple clinical trials have preliminarily explored the potential of Tislelizumab in LC treatment, demonstrating certain ORR and DCR in monotherapy or combination therapy for locally advanced or metastatic LC (Zhao et al., 2024; Daei Sorkhabi et al., 2023; Lu et al., 2024). However, variations among studies in patient populations, treatment lines, combination regimens, and control group selection have led to inconsistencies in the exact efficacy and safety data, leaving the clinical positioning of Tislelizumab in such patients still controversial.

In the absence of direct head-to-head RCTs comparing tislelizumab with other established immunotherapies, indirect treatment comparisons (ITCs) have emerged as a crucial methodological approach to inform clinical decision-making. ITCs, including network meta-analyses and adjusted indirect comparisons, allow for the estimation of relative treatment effects by leveraging common comparator arms across different trials (e.g., chemotherapy alone). Recent ITCs have specifically evaluated tislelizumab versus pembrolizumab, both combined with chemotherapy, as first-line treatment for advanced NSCLC. For instance, a systematic review and indirect comparison by Guo et al. (2023) found no significant differences in PFS (HR = 1.04, 95% CI: 0.82–1.31), ORR (RR = 0.79, 95% CI: 0.59–1.07), or incidence of grade ≥3 Adverse event (AE) (RR = 0.99, 95% CI: 0.87–1.12) between tislelizumab and pembrolizumab combinations, suggesting comparable efficacy and safety profiles. Similarly, Messori et al. (2023) applied the IPDfromKM-Shiny method to reconstruct individual patient data from Kaplan-Meier curves of five RCTs and reported substantial equivalence in PFS between the two regimens (HR = 0.952, 95% CI: 0.775–1.168). These findings were further supported by a real-world retrospective cohort study in the neoadjuvant setting, which demonstrated no significant differences in pathological response rates, survival outcomes, or toxicity profiles between pembrolizumab and tislelizumab when combined with chemotherapy (Hu et al., 2025). Given the consistent results across these methodological approaches, ITCs provide robust supplementary evidence for positioning tislelizumab relative to other immunotherapies, highlighting its non-inferior efficacy and safety in advanced LC contexts.

To comprehensively and objectively evaluate the therapeutic effects and safety of Tislelizumab in locally advanced or metastatic LC, we systematically searched for relevant RCTs and single-arm studies up to 19 May 2025, and conducted a meta-analysis based on the PRISMA guidelines. This study aims to integrate existing evidence, focusing on comparing the differences between Tislelizumab and standard treatment or placebo in terms of ORR, DCR, OS, and PFS, and to explore its performance in different subtypes of LC, thereby providing more reliable evidence-based medical support for clinical decision-making.

## 2 Materials and methods

This protocol has been registered in the International Prospective Register of Systematic Reviews (PROSPERO: CRD420251069321).

### 2.1 Search strategy

We searched PubMed, Embase, Cochrane Library, and Web of Science for articles published up to 19 May 2025, on the efficacy and safety of Tislelizumab in treating locally advanced or metastatic LC. The search terms were (Lung Cancer, Nasopharyngeal, nasopharyngeal cancer, NPC) AND (tislelizumab, BGB-A317). The specific search strategies for PubMed and Embase are provided in Supplementary Tables S1, S2.

### 2.2 Inclusion and exclusion criteria

Inclusion criteria (Abu Rous et al., 2023): Study design: RCTs or single-arm studies (Allemani et al., 2018); Study population: patients with locally advanced or metastatic LC (including NSCLC and SCLC) diagnosed by histology or cytology (Frödin, 1996); Intervention: the study group received Tislelizumab (either as monotherapy or in combination with other therapies) as a treatment regimen (Sullivan, 2018); Comparator: For RCTs, the control group received one of the following standard-of-care comparators: a. Placebo plus chemotherapy. b. alone (e.g., platinum-based doublets such as carboplatin/paclitaxel, cisplatin/pemetrexed). c. Active drugs (e.g., docetaxel as second-line therapy) (Li et al., 2023). For single-arm studies, the efficacy of Tislelizumab was evaluated against historical benchmarks or within the study cohort without a direct concurrent control group (Legha et al., 1977). Outcome measures: studies reporting at least one of the following outcomes: OS, PFS, ORR [defined as the sum of complete response (CR) and partial response (PR)], or DCR [defined as the sum of CR, PR, and stable disease (SD)], or treatment-related adverse events (TRAEs).

Exclusion criteria (Abu Rous et al., 2023): Document type: duplicate publications, conference abstracts, literature reviews, meta-analyses, or case reports (Allemani et al., 2018); Study relevance: studies unrelated to the treatment of locally advanced or metastatic LC with Tislelizumab (Frödin, 1996); Data integrity: studies from which complete data or required outcome measures (such as OS, PFS, ORR, DCR, TRAEs) could not be obtained.

### 2.3 Data extraction

Extracted data included author names, publication year, drug type, number of included cases, drug dosage, follow-up duration, median OS, median PFS, median ORR, median DCR, TRAEs, and basic study information. Data extraction was independently performed by two researchers.

### 2.4 Risk of bias assessment

For RCTs: the Cochrane Risk of Bias tool for randomized trials 2.0 (RoB2) was used to assess the risk of bias (Sterne et al., 2019). RoB2 was applied by two independent researchers, and a third researcher resolved any disagreements in bias risk assessment. Evaluators examined the randomization process, deviations from intended interventions, missing outcome data, selection of outcome measures, and reported outcomes. Thus, studies were categorized as having low, moderate, or high risk of bias. For single-arm studies: the Methodological Index for Non-Randomized Studies (MINORS) (Slim et al., 2003) was used to assess quality, with the following grading: 0–12 points: low quality; 13–18 points: moderate quality; 19–24 points: high quality. Any disagreements were resolved through consultation (Supplementary Table S3).

### 2.5 Data analysis

For randomized controlled trials, we analyzed binary variables such as ORR, DCR, PFS, and OS using OR and 95% CI. For single-arm studies, we used effect sizes (ES) and 95% CI. Due to substantial heterogeneity in treatment types, frequencies, and durations across studies, a random-effects model was employed for the meta-analysis. Statistical analysis was performed using Stata software (version 15.0; Stata Corp, College Station, TX, United States). Heterogeneity was assessed using I^2^ values or Q statistics. I^2^ values of 0%, 25%, 50%, and 75% indicated no, low, moderate, and high heterogeneity, respectively. When I^2^ ≥ 50%, a sensitivity analysis was conducted to investigate potential sources of heterogeneity; otherwise, a fixed-effects model was applied. Additionally, publication bias was evaluated using Egger’s test or Begg’s test with a random-effects model. A two-sided p < 0.05 was considered statistically significant.

## 3 Results

### 3.1 Literature screening and characteristics

**FIGURE 2:**
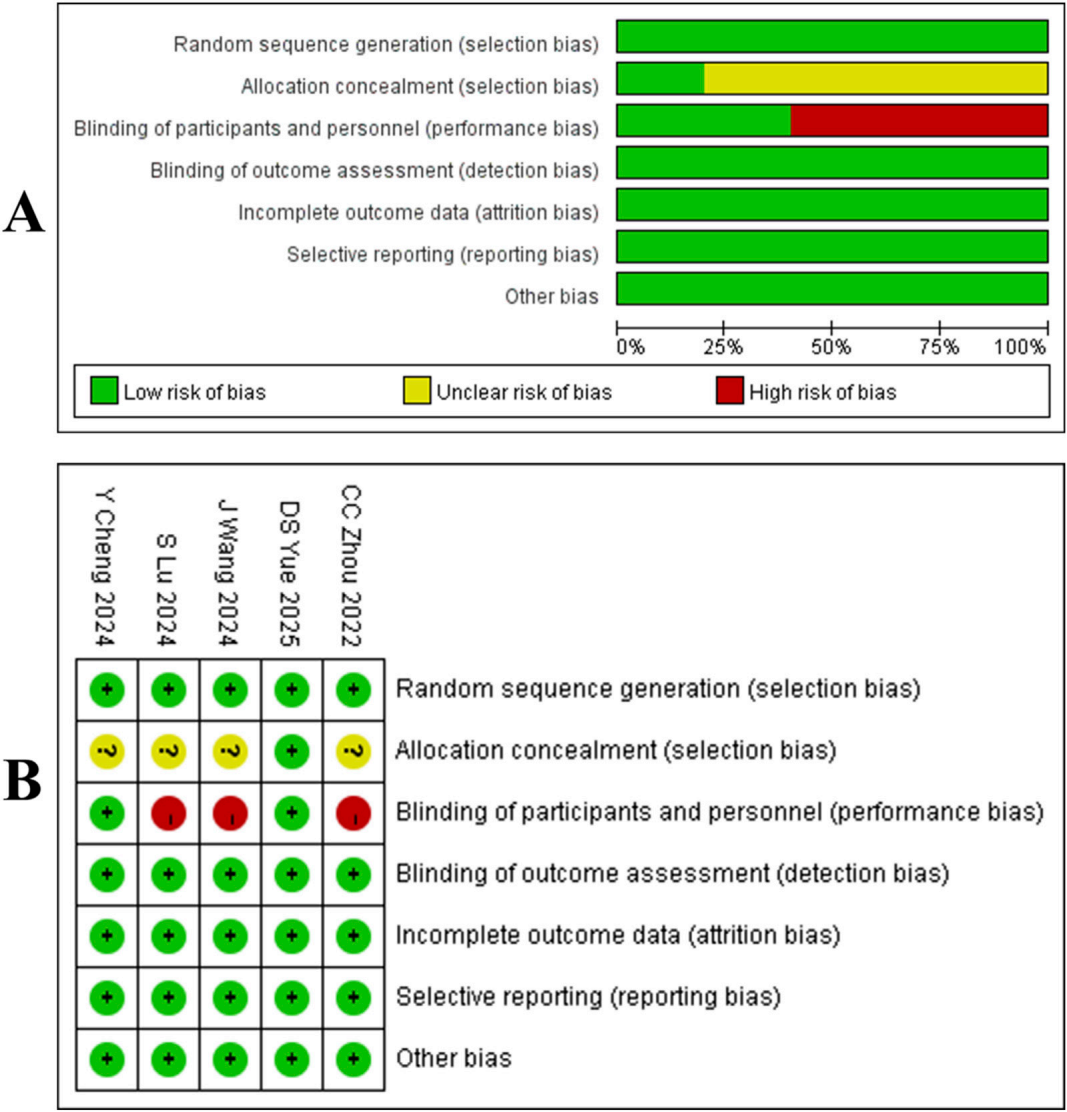
**(A)** Quality assessment results of included literature. **(B)** three risk levels are intuitively represented using a combination of colors and symbols: Low risk: Indicated by a green circle containing a plus sign (+). Some concerns: Indicated by a yellow circle containing a question mark (?). High risk: Indicated by a red circle containing a question mark (?).

**FIGURE 3:**
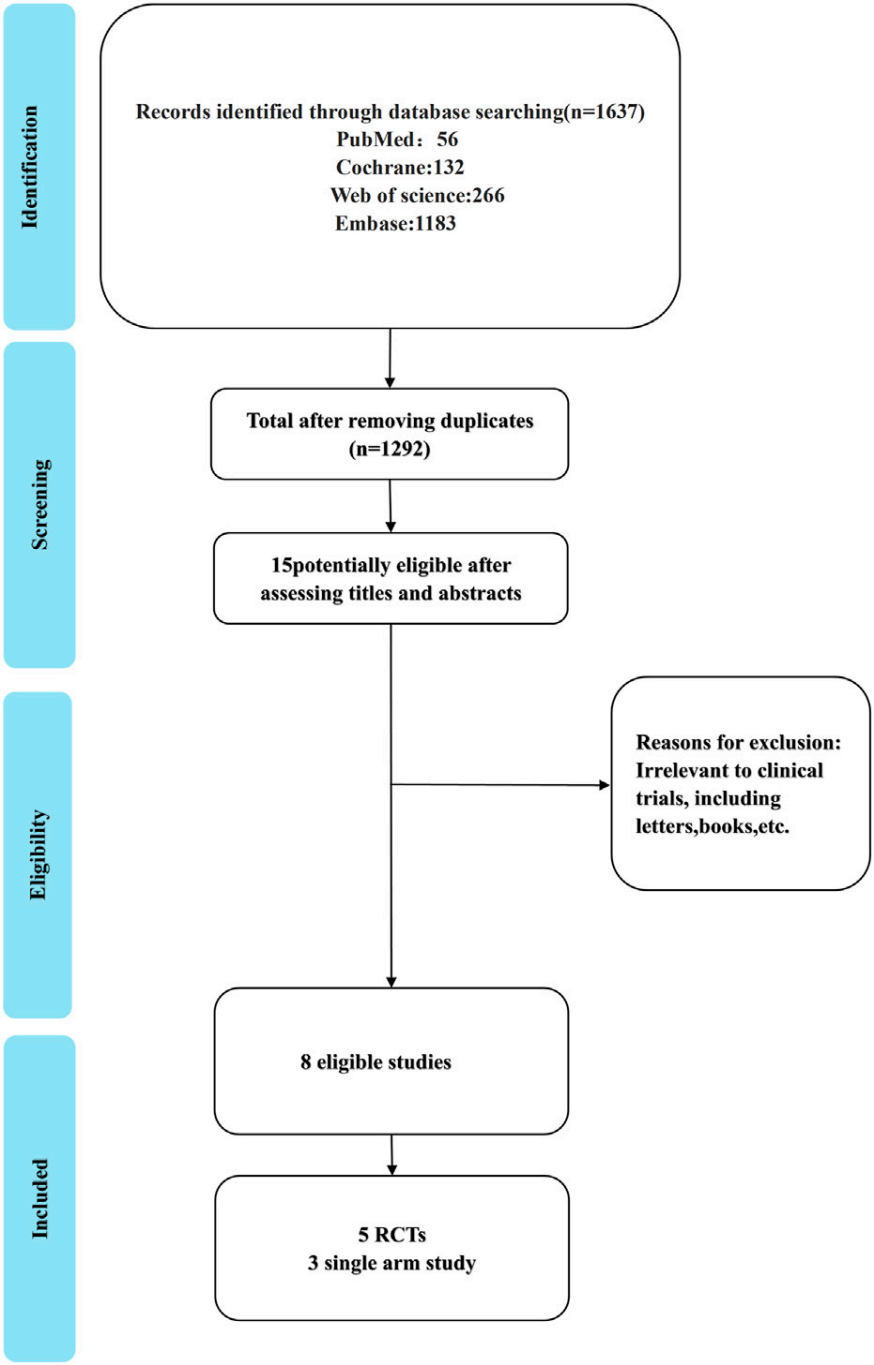
Process and outcomes of literature screening.

Our search yielded 1,637 articles, of which 1,292 remained after duplicate removal. A detailed review of titles and abstracts narrowed down the selection to 15 articles, and upon thorough full-text evaluation, we included 8 high-quality studies (Cheng Y. et al., 2024; Lu et al., 2024; Zhou et al., 2023; Wang J. et al., 2024; Yue et al., 2025; Zhu et al., 2022; Zhao et al., 2023; Wang Z. et al., 2021; Figure 2). Five of these were RCTs (Cheng Y. et al., 2024; Lu et al., 2024; Zhou et al., 2023; Wang J. et al., 2024; Yue et al., 2025), encompassing 2,290 participants with 1,331 in the Tislelizumab group and 959 in the control group, the PICO (Population, Intervention, Comparison, Outcomes) characteristics of these RCTs are summarized in Table 1. The remaining three were single-arm studies (Zhu et al., 2022; Zhao et al., 2023; Wang Z. et al., 2021) with 115 participants. Notably, one RCT (Yue et al., 2025) did not provide data on median ORR, median OS, median PFS, or median DCR, but it did report TRAES, and thus it was included in our analysis (Figure 3; Table 2).

**TABLE 1:** PICO characteristics of the included RCTs.

Study	Population	Intervention	Comparison	Outcomes reported
Cheng et al. (2024b)	Adults with extensive-stage SCLC	Tislelizumab + Platinum + Etoposide	Placebo + Platinum + Etoposide	OS, PFS, ORR, DCR, TRAEs
Lu et al. (2024)	Adults with locally advanced or metastatic non-squamous NSCLC	Tislelizumab + Pemetrexed + Platinum	Placebo + Pemetrexed + Platinum	OS, PFS, ORR, DCR, TRAEs
Zhou et al. (2023)	Adults with previously treated advanced NSCLC	Tislelizumab (monotherapy)	Docetaxel	OS, PFS, ORR, DCR, TRAEs
Wang et al. (2024a)	Adults with advanced squamous NSCLC	Tislelizumab + Paclitaxel/Nab-paclitaxel + Carboplatin	Placebo + Paclitaxel/Nab-paclitaxel + Carboplatin	OS, PFS, ORR, DCR, TRAEs
Yue et al. (2025)	Adults with resectable NSCLC (perioperative)	Perioperative Tislelizumab + Neoadjuvant Chemotherapy	Neoadjuvant Chemotherapy alone	pCR, MPR, EFS, TRAEs

Abbreviations: SCLC: small cell lung cancer; NSCLC: non-small cell lung cancer; OS: overall survival; PFS: progression-free survival; ORR: objective response rate; DCR: disease control rate; TRAEs: treatment-related adverse events; pCR: pathological complete response; MPR: major pathological response; EFS: event-free survival.

**TABLE 2:** Literature baseline table.

Study	Ref.	Study type	Cancer type	Method of administration of tislelizumab		Sample size (male)		Mean age (years)		Follow up (Mo)	Median OS(Mo)		Median PFS (Mo)		Median ORR (%)		Median DCR (%)		TNM stage	PD-L1 ≥ 50%(n)		Metastatic sites
				Dose (mg)	Frequency	T	C	T	C		T	C	T	C	T	C	T	C		T	C	
Cheng et al. (2024b)	33	RCT	SCLC	200	Q3W	227 (186)	230 (186)	62	62	14.2	15.5	13.5	4.7	4.3	68	62	89	88	IIIA, IIIB, IV	NR	NR	Liver, Brain
Lu et al. (2024)	34	RCT	NSCLC	200	Q3W	223 (168)	111 (79)	60	61	16.1	21.4	21.3	9.7	5.6	57.4	36	59.2	81.1	IIIB, IV	36	42	Bone, Liver, Brain
Zhou et al. (2023)	40	RCT	NSCLC	200	Q3W	535 (416)	270 (260)	61	61	16	17.2	11.9	4.2	2.6	22.6	7.1	55.7	42.2	NR	NR	74	Bone, Liver, Brain
Wang et al. (2024a)	41	RCT	NSCLC	200	Q3W	120 (107)	121 (111)	60	63	16.7	22.8	20.2	9.6	5.5	70	49.6	92.5	86.8	IIIB, IV	41	NR	Bone, Liver, Brain
Yue et al. (2025)	42	RCT	NSCLC	200	Q3W	226 (205)	227 (205)	63	62	22	NRE	NRE	NR	NR	NR	NR	NR	NR	II, IIIA	71	62	NR
Zhu et al. (2022)	43	SAE	NSCLC	200	Q3W	29 (14)		70.3		14	16.5		9.5		34.5		86.2		NR	NR		Bone, Liver, Brain, Lung
Zhao et al. (2023)	44	SAE	NSCLC	200	Q3W	69 (38)		58		8.2	NRE		7.6		56.5		87.1		IIIB, IV	NR		Liver, Bone, CNS
Wang et al. (2021a)	45	SAE	SCLC	200	Q3W	17 (13)		60		NR	15.6		6.9		77		88		III, IV	NR		Lung, Bone, Liver, Brain

Reference; RCT: random control study; SAE: single-armed experiment; T: trails group; C: control group; NSCLC: small cell lung cancer; SCLC: small cell lung cancer; MO: months; OS: overall survival; PFS: progression free survival; ORR: objective response rate; DCR: disease control rate; PD-L1: programmed cell death ligand-1; NR: not reported; NRE: not reached; CNS: central nervous system; Q3W: every 3 weeks.

### 3.2 Meta-analysis of RCTs

#### 3.2.1 ORR

**FIGURE 4:**
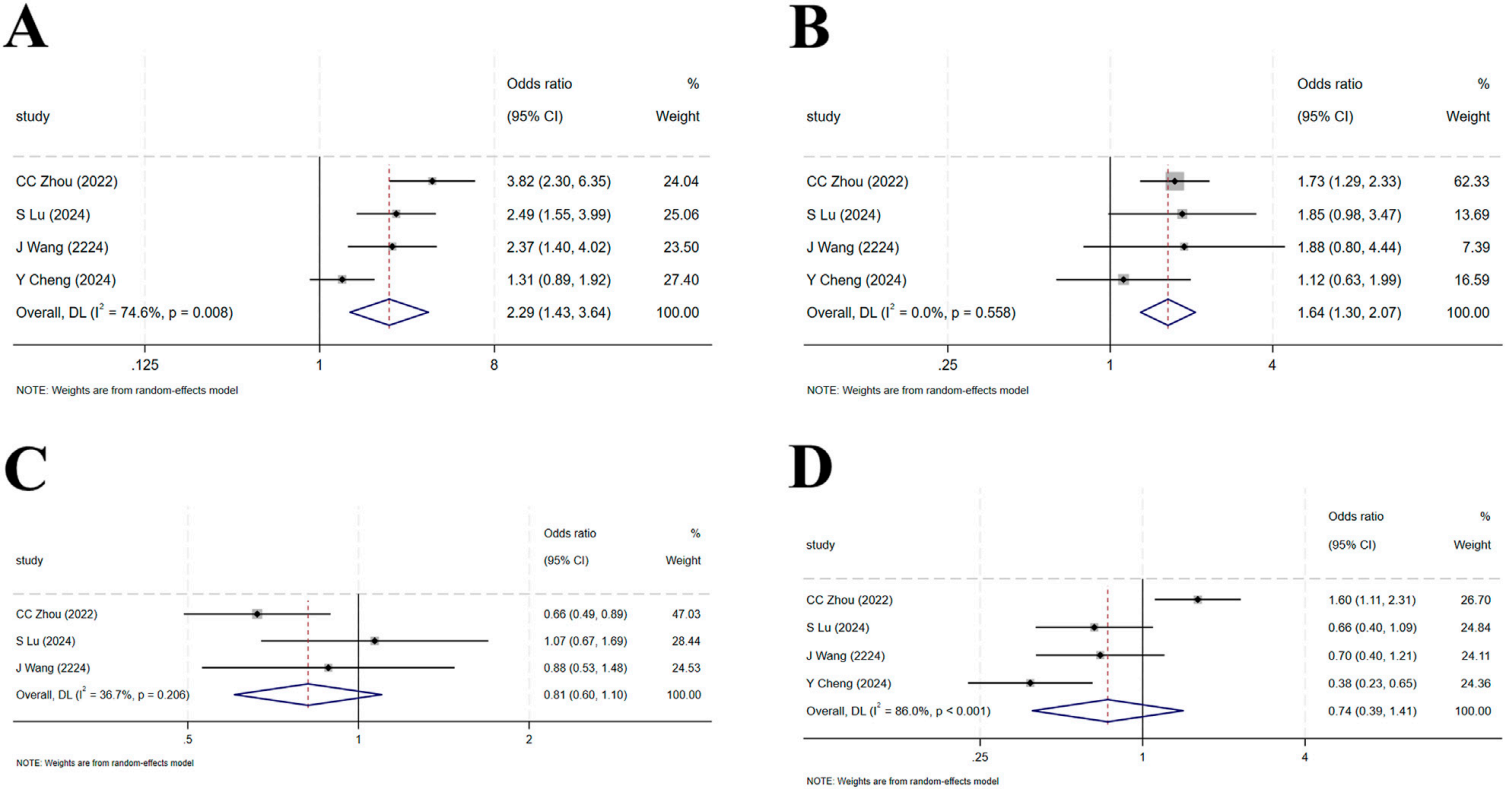
**(A)** Forest plot of overall response rate (ORR) in RCTs; **(B)** Forest plot of disease control rate (DCR) in RCTs; **(C)** Forest plot of overall survival (OS) in RCTs; **(D)** Forest plot of progression-free survival (PFS) in RCTs.

Four studies (Cheng Y. et al., 2024; Lu et al., 2024; Zhou et al., 2023; Wang J. et al., 2024) enrolled a total of 1,837 patients with locally advanced or metastatic LC, including 1,105 in the tislelizumab group and 732 in the control group (I^2^ = 74.6%, p = 0.008), indicating high heterogeneity. The forest plot showed [OR = 2.29, 95%CI (1.43, 3.64), p = 0.001],suggesting that tislelizumab significantly improved the ORR in these patients (Figure 4A; Table 2). Sensitivity analysis, conducted by sequentially excluding studies, indicated that potential heterogeneity may stem from Cheng Y. et al. (2024) (Supplementary Figure S1A). Publication bias was assessed using Egger’s test (p = 0.122) and Begg’s test (p = 0.497), both p-values >0.05, suggesting a low likelihood of publication bias (Supplementary Table S4).

#### 3.2.2 DCR

Four studies (Cheng Y. et al., 2024; Lu et al., 2024; Zhou et al., 2023; Wang J. et al., 2024) included 1,837 patients, with 1,105 in the tislelizumab group and 732 in the control group (I^2^ = 0, p = 0.558), indicating low heterogeneity. The forest plot showed [OR = 1.64, 95%CI (1.30, 2.07), p < 0.001], demonstrating that tislelizumab significantly improved the DCR in patients with locally advanced or metastatic LC (Figure 4B; Table 2). Publication bias was evaluated using Egger’s test (p = 0.806) and Begg’s test (p = 0.497), both p-values >0.05, indicating a low likelihood of publication bias (Supplementary Table S4).

#### 3.2.3 OS

Four studies (Cheng Y. et al., 2024; Lu et al., 2024; Zhou et al., 2023; Wang J. et al., 2024) involved 1,837 patients, with 1,105 in the tislelizumab group and 732 in the control group (I^2^ = 36.7%, p = 0.206), suggesting moderate heterogeneity. The forest plot showed [OR = 0.81, 95%CI (0.60, 1.10), p = 0.179], suggesting that tislelizumab did not improve the OS in patients with locally advanced or metastatic LC (Figure 4C; Table 2). Publication bias was assessed using Egger’s test (p = 0.309) and Begg’s test (p = 0.602), both p-values >0.05, indicating a low likelihood of publication bias (Supplementary Table S4).

#### 3.2.4 PFS

Four studies (Cheng Y. et al., 2024; Lu et al., 2024; Zhou et al., 2023; Wang J. et al., 2024) included 1,380 patients, with 878 in the tislelizumab group and 502 in the control group (I^2^ = 86.0%, p < 0.001), indicating high heterogeneity. The forest plot showed [OR = 0.74, 95%CI (0.39, 1.41), p = 0.364], indicating that tislelizumab did not improve the OS in patients with locally advanced or metastatic LC (Figure 4D; Table 2). Sensitivity analysis by sequentially removing studies revealed that potential heterogeneity may originate from C Zhou (2023) (Zhou et al., 2023; Supplementary Figure S1B). The p-values of Egger’s test (p = 0.079) and Begg’s test (p = 0.497) were both >0.05, indicating a low likelihood of publication bias (Supplementary Table S4).

### 3.3 Meta-analysis of single-arm studies

#### 3.3.1 ORR

**FIGURE 5:**
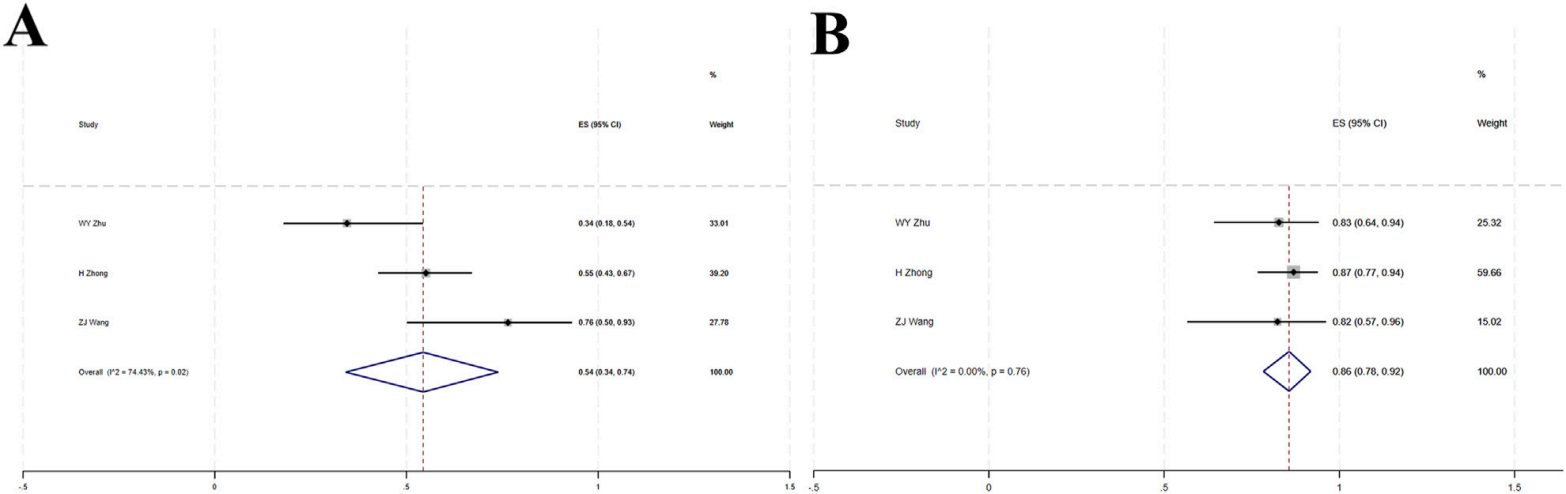
**(A)** Forest plot of overall response rate (ORR) in single-arm studies; **(B)** Forest plot of disease control rate (DCR) in single-arm studies.

Three studies (Zhu et al., 2022; Zhao et al., 2023; Wang Z. et al., 2021) involved a total of 115 patients with locally advanced or metastatic LC. High heterogeneity was observed among the studies (I^2^ = 74.43%, p = 0.02). The forest plot showed [OR = 0.54, 95%CI (0.34, 0.74), p < 0.001], indicating that tislelizumab improved the ORR in these patients (Figure 5A; Table 2). Sensitivity analysis suggested that potential heterogeneity may stem from the study by Zhu et al. (2022) (Supplementary Figure S2). Assessment of publication bias using Egger’s test (p = 0.873) and Begg’s test (p = 0.602) yielded p-values >0.05, indicating a low likelihood of publication bias (Supplementary Table S4).

#### 3.3.2 DCR

Three studies (Zhu et al., 2022; Zhao et al., 2023; Wang Z. et al., 2021) included a total of 115 patients with locally advanced or metastatic LC. Low heterogeneity was observed (I^2^ = 0, p = 0.76). The forest plot showed [OR = 0.86, 95%CI (0.78, 0.92), p < 0.001], indicating that tislelizumab improved the DCR in these patients (Figure 5B; Table 2). Assessment of publication bias using Egger’s test (p = 0.209) and Begg’s test (p = 0.602) yielded p-values >0.05, indicating a low likelihood of publication bias (Supplementary Table S4).

### 3.4 Subgroup analysis

Subgroup analyses were conducted based on the histological subtypes of LC.

#### 3.4.1 ORR

**FIGURE 6:**
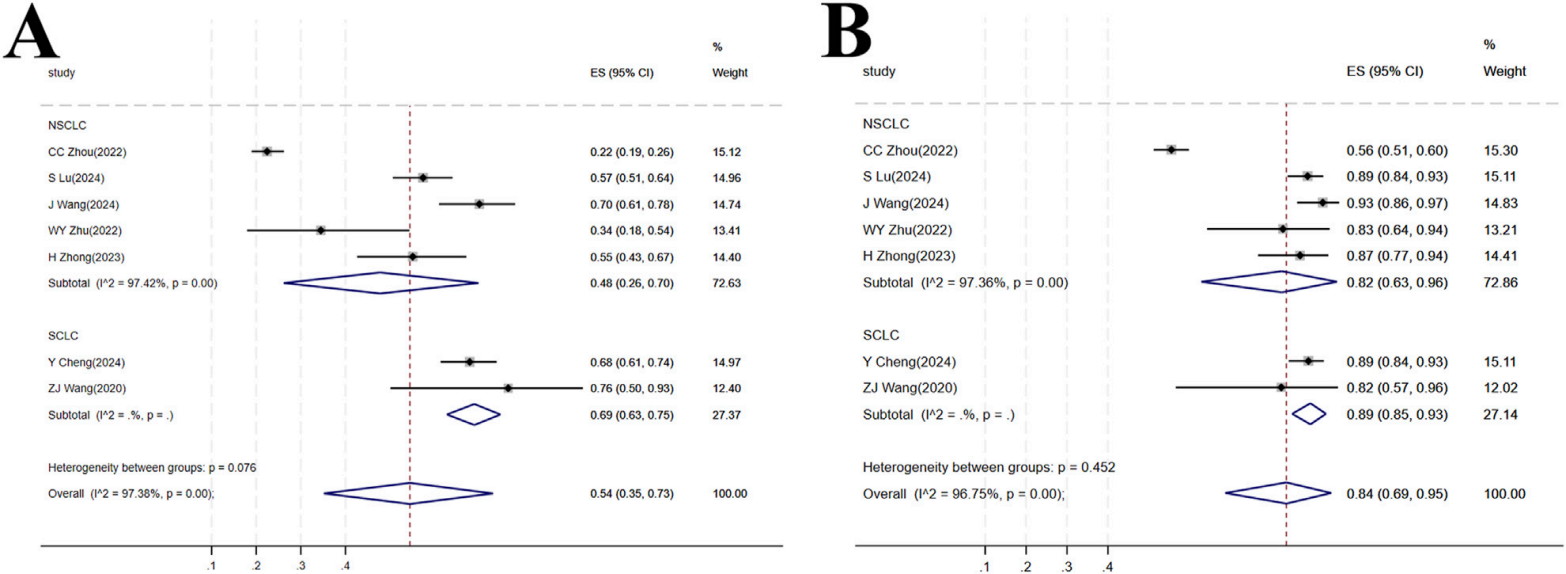
**(A)** Forest plot of overall response rate (ORR) in subgroup analyses; **(B)** Forest plot of disease control rate (DCR) in subgroup analyses.

Seven studies (Cheng Y. et al., 2024; Lu et al., 2024; Zhou et al., 2023; Wang J. et al., 2024; Zhu et al., 2022; Zhao et al., 2023; Wang Z. et al., 2021) involving a total of 547 patients were included. This population comprised 380 patients with NSCLC from five studies (Lu et al., 2024; Zhou et al., 2023; Wang J. et al., 2024; Zhu et al., 2022; Zhao et al., 2023) and 167 patients with SCLC from two studies. In the NSCLC subgroup, high heterogeneity was observed among the studies (I^2^ = 97.42%, p < 0.001), and the forest plot showed [OR = 0.48, 95%CI (0.26, 0.74), p < 0.001], suggesting that tislelizumab improved the ORR in patients with NSCLC. For the SCLC subgroup, [OR = 0.69, 95%CI (0.63, 0.75), p < 0.001] indicated that tislelizumab improved the ORR in patients in the SCLC subgroup. The heterogeneity between the two groups for tislelizumab was not significant (p = 0.076), indicating that the effect of tislelizumab was similar in the two subgroups (Figure 6A; Table 2).

#### 3.4.2 DCR

The same seven studies (Cheng Y. et al., 2024; Lu et al., 2024; Zhou et al., 2023; Wang J. et al., 2024; Zhu et al., 2022; Zhao et al., 2023; Wang Z. et al., 2021) including 547 patients were analyzed for DCR. The NSCLC subgroup consisted of 380 patients from five studies (Lu et al., 2024; Zhou et al., 2023; Wang J. et al., 2024; Zhu et al., 2022; Zhao et al., 2023) while the SCLC subgroup comprised 167 patients from two studies (Cheng Y. et al., 2024; Wang Z. et al., 2021). High heterogeneity was present within the NSCLC subgroup (I^2^ = 97.36%, p < 0.001). The forest plot showed [OR = 0.82, 95%CI (0.63, 0.96), p < 0.001], indicating that tislelizumab improved the DCR in this patient group. In the SCLC subgroup, [OR = 0.89, 95%CI (0.85, 0.93), p < 0.001], suggesting an improvement in DCR with tislelizumab. The non-significant heterogeneity between the subgroups (p = 0.452) implied a comparable effect of tislelizumab on DCR in both NSCLC and SCLC patients (Figure 6B; Table 2).

### 3.5 TRAES

A meta-analysis was conducted on TRAES reported in the 8 included studies (Cheng Y. et al., 2024; Lu et al., 2024; Zhou et al., 2023; Wang J. et al., 2024; Yue et al., 2025; Zhu et al., 2022; Zhao et al., 2023; Wang Z. et al., 2021). The analysis focused on events reported in at least two studies. Common AEs included increased Alanine Aminotransferase (ALT), increased Aspartate Aminotransferase (AST), anemia, decreased appetite, nausea, decreased neutrophil count, decreased platelet count, rash, decreased white blood cell count, leukopenia, hypothyroidism, and hyponatremia. The incidence rates for these events were as follows: increased ALT (18.87%), increased AST (13.62%), anemia (32.15%), decreased appetite (10.92%), nausea (16.18%), decreased neutrophil count (23.78%), decreased platelet count (10.16%), rash (5.4%), decreased white blood cell count (26.55%), leukopenia (10.23%), hypothyroidism (8.36%), and hyponatremia (4.19%). Regarding events of Grade 3 or higher, the rates were: increased ALT (0.89%), increased AST (0.62%), anemia (4.70%), decreased appetite (0.20%), nausea (0.13%), decreased neutrophil count (17.15%), decreased platelet count (1.79%), rash (0.69%), decreased white blood cell count (8.71%), leukopenia (3.18%), hypothyroidism (0.20%), and hyponatremia (0.34%). Constipation was also observed as a common AE, with an overall incidence of 8.50%, although no Grade 3 or higher events related to constipation were reported (Table 3).

**TABLE 3:** Results of meta-analysis of adverse events.

Adverse event	Any grade				Grade ≥3			
	Study	Heterogeneity		ES (95%CI)	Study	Heterogeneity		ES (95%CI)
		P	I^2^ (%)			P	I^2^ (%)	
ALT increased	5	<0.001	85.42	0.25 (0.18,0.33)	5	0.88	<0.001	0.01 (0.00,0.02)
AST increased	4	0.01	75.44	0.20 (0.15,0.26)	3	0.71	<0.001	0.01 (0.00,0.02)
Anemia	6	<0.001	99.01	0.44 (0.16,0.74)	5	0	94.32	0.07 (0.02,0.15)
Decreased appetite	5	<0.001	96.03	0.20 (0.08,0.37)	3	0.68	<0.001	0.00 (0.00,0.01)
Fatigue	3	0.27	24.00	0.11 (0.08,0.14)	3	0.29	19.01	0.01 (0.00,0.2)
Nausea	5	<0.001	97.46	0.24 (0.10,0.42)	2	0	<0.001	0.00 (0.00,0.01)
Neutrophil count decreased	5	<0.001	99.43	0.39 (0.07,0.78)	5	0	99.29	0.24 (0.02,0.60)
Neutropenia	3	<0.001	99.63	0.32 (0.00,0.87)	2	0	<0.001	0.48 (0.43,0.53)
Platelet count decreased	4	<0.001	91.10	0.26 (0.15,0.39)	4	0.06	58.57	0.04 (0.02,0.07)
Rash	4	0.40	0	0.14 (0.11,0.17)	3	0.95	<0.001	0.02 (0.01,0.03)
Thrombocytopenia	2	<0.001	0	0.42 (0.37,0.47)	2	0	<0.001	0.14 (0.11,0.18)
White blood cell count decreased	5	<0.001	99.32	0.43 (0.11,0.78)	5	0	98.01	0.12 (0.02,0.29)
Lymphocyte count decreased	2	<0.001	0	0.12 (0.08,0.16)	2	0	<0.001	0.04 (0.02,0.06)
Leukopenia	4	<0.001	98.67	0.19 (0.01,0.50)	3	0.16	46.29	0.11 (0.07,0.17)
Hypothyroidism	4	0.36	6.02	0.12 (0.10,0.14)	2	0	<0.001	0.01 (0.00,0.02)
Hyponatremia	4	<0.001	87.11	0.12 (0.04,0.22)	2	0	<0.001	0.01 (0.00,0.02)
Pain in extremity	2	<0.001	0	0.16 (0.13,0.21)	1	NR	NR	0.03 (0.01,0.07)
Alopecia	5	<0.001	99.51	0.41 (0.06,0.82)	1	NR	NR	0.00 (0.00,0.02)
Asthenia	3	<0.001	88.91	0.13 (0.05,0.24)	1	NR	NR	0.00 (0.00,0.01)
Vomiting	3	<0.001	86.19	0.16 (0.09,0.25)	1	NR	NR	0.00 (0.00,0.02)
Diarrhe	2	<0.001	0	0.03 (0.02,0.05)	1	NR	NR	0.00 (0.00,0.01)
Hypercholesterolemia	2	<0.001	0	0.23 (0.15,0.33)	NR			
Constipation	5	<0.001	95.71	0.13 (0.05,0.24)	NR			
Poesthesia	3	0.88	0.00	0.20 (0.16,0.24)	NR			

Abbreviation: CI: confidence interval; NR: not reported.

## 4 Discussion

### 4.1 Summary of main efficacy findings

Compared with chemotherapy, PD-L1 inhibitors have been reported to prolong PFS and OS, particularly in patients with high tumor PD-L1 expression (Mok et al., 2019; Jassem et al., 2021; Sezer et al., 2021; Herbst et al., 2020). Several global trials have demonstrated that anti-PD-1 or anti-PD-L1 therapy provides significant efficacy and safety advantages for advanced LC (Borghaei et al., 2015; Barlesi et al., 2018; Herbst et al., 2016; Brahmer et al., 2015; Rittmeyer et al., 2017). This study, through a systematic review and meta-analysis, aims to integrate existing evidence and evaluate the efficacy and safety of Tislelizumab in the treatment of locally advanced or metastatic LC. The comprehensive analysis results indicate that Tislelizumab shows positive potential in improving short-term efficacy indicators in patients, especially in ORR and DCR, but its impact on long-term survival benefits still requires more high-quality data for confirmation.

### 4.2 Mechanistic rationale for short-term efficacy and Fc engineering advantage

First, regarding efficacy, we observed that in the RCT subgroup, Tislelizumab significantly increased the ORR [OR = 2.29, 95%CI (1.43,3.64), P = 0.001] and DCR [OR = 1.64, 95%CI (1.30,2.07), P < 0.001] of patients with locally advanced or metastatic LC compared with the control group (Cheng Y. et al., 2024; Lu et al., 2024; Zhou et al., 2023; Wang J. et al., 2024; Yue et al., 2025). This finding is consistent with Tislelizumab’s mechanism as a PD-1 inhibitor, which aims to relieve T-cell inhibition and restore anti-tumor immune responses, suggesting that it can effectively induce tumor regression or stabilization (Lin et al., 2025; Dammeijer et al., 2020; Kumagai et al., 2020). Furthermore, the distinct structural design of Tislelizumab is hypothesized to contribute to its efficacy profile. Unlike some conventional anti-PD-1 antibodies, Tislelizumab is engineered with a specific Fc domain modification that minimizes binding to Fcγ receptors (FcγR) on macrophages and other myeloid cells (Zhao et al., 2024; Cheng Y. et al., 2024; Dahan et al., 2015). This design is crucial because binding of an anti-PD-1 antibody’s Fc domain to activating FcγRs (e.g., FcγRIIIa) can trigger ADCP of T cells expressing PD-1, paradoxically depleting the very immune effector cells intended to be activated (Dahan et al., 2015; Arlauckas et al., 2017). By mitigating this Fc-mediated effector function, Tislelizumab may potentially preserve the tumor-infiltrating T-cell pool, leading to a more robust and sustained anti-tumor immune response compared to antibodies capable of inducing significant ADCP (Zhang et al., 2018; Lee et al., 2017). This theoretical advantage in the tumor microenvironment could provide a mechanistic rationale for the significant improvements in short-term efficacy endpoints (ORR and DCR) observed in our pooled analysis. However, it is important to note that direct comparative clinical data confirming this mechanistic superiority over other PD-1 inhibitors remain limited, and the impact of this design on long-term survival outcomes requires further validation in well-controlled studies.

### 4.3 Interpretation of long-term survival outcomes and potential explanations

However, for the more critical long-term survival indicators, namely, OS and PFS, the RCT subgroup analysis did not show a statistically significant increase OS [OR = 0.81, 95%CI (0.60,1.10), P = 0.179]; PFS [OR = 0.74, 95%CI (0.39,1.41), P = 0.364]. Although the OR value of OS is less than 1, indicating a potential survival benefit trend, which is basically consistent with the report by Zhang et al. (2022) the confidence interval includes the null value, and PFS also did not reach significance, suggesting that the current evidence based on RCTs is insufficient to confirm that Tislelizumab can provide definite long-term survival advantages. A doubt worth further exploration is why Tislelizumab performs well in short-term indicators (ORR/DCR) but fails to show statistical advantages in OS/PFS, which reflect long-term benefits. This may not mean that Tislelizumab is ineffective, but rather reflects potential limitations in study design or execution. For example, the included RCTs may have relatively limited sample sizes, resulting in insufficient statistical power to detect true differences in OS/PFS; or, the follow-up time may not be long enough to fully capture the long-term survival improvement effect of the treatment. Additionally, factors such as the choice of the control group (e.g., placebo or active drug), heterogeneity in baseline patient characteristics, and tumor type (e.g., NSCLC or SCLC, although the abstract did not clearly distinguish, but different subtypes exhibit significant differences in response patterns to immunotherapy) may also affect the between-group comparison results of OS/PFS. Heterogeneity analysis suggests that differences between some studies may have affected the stability of the pooled effect size, such as the contribution of Cheng Y. et al. (2024) to the heterogeneity of ORR and the influence of Zhou et al. (2023) on the heterogeneity of PFS, which also reflects potential differences in baseline patient characteristics, treatment lines, or combination regimens among different studies to some extent. Therefore, we are cautious about the conclusion that Tislelizumab did not significantly improve OS/PFS, believing that this reflects more the strength of current evidence rather than a final negation of its long-term value.

### 4.4 Critical appraisal of single-arm evidence and its limitations

On the other hand, data from single-arm studies offer additional insights into the use of Tislelizumab (Zhu et al., 2022; Zhao et al., 2023; Wang Z. et al., 2021). Although single-arm designs limit the ability to make direct comparisons with control groups, their findings also indicate favorable outcomes associated with Tislelizumab treatment (Iaquinto et al., 2025; Wang M. et al., 2024). The pooled ORR [OR = 0.54, 95%CI (0.34–0.74), P < 0.001] and DCR [OR = 0.86, 95%CI (0.78–0.92), P < 0.001] from these single-arm studies were both substantial, suggesting that Tislelizumab monotherapy or specific regimens can achieve meaningful clinical responses and disease control even without a direct comparator. This further underscores the potential value of Tislelizumab as a treatment option, particularly for patient populations who are ineligible or unsuitable for randomized controlled trials (Wang J. et al., 2021; Wang L. et al., 2024).

However, the interpretation of efficacy results from single-arm studies requires considerable caution due to inherent methodological limitations. The primary constraint is the absence of a concurrent control group, which makes it impossible to attribute observed outcomes (e.g., high ORR and DCR) solely to the investigational intervention (Booth and Tannock, 2014; Bothwell and Podolsky, 2016). Without randomization, significant biases can influence the results. These include selection bias, as patients enrolled in single-arm trials may not be representative of the broader patient population due to strict eligibility criteria; performance bias and detection bias, as the open-label design can influence both the administration of care and the assessment of outcomes; and confounding by unknown prognostic factors (Sacks et al., 1982; Vetter and Mascha, 2017). The favorable outcomes reported in the single-arm studies of Tislelizumab (Zhu et al., 2022; Zhao et al., 2023; Wang Z. et al., 2021) could potentially be influenced by these biases. Therefore, while these results are promising and suggest clinical activity, they cannot establish causal efficacy or provide a robust estimate of the magnitude of benefit relative to a standard of care or placebo. The data from single-arm studies are best interpreted as generating hypotheses and providing preliminary evidence of activity, which must then be confirmed in well-designed randomized controlled trials (Owzar, 2008; Fogel, 2018). Consequently, the pooled ORR and DCR from our single-arm analysis, though statistically significant, should be viewed as supportive rather than conclusive evidence of Tislelizumab’s efficacy.

### 4.5 Efficacy across histological subtypes: NSCLC versus SCLC

Subgroup analysis yielded interesting results, indicating that Tislelizumab may exhibit similar efficacy in improving ORR and DCR across the two major LC subtypes: NSCLC and SCLC (Cheng Y. et al., 2024; Lu et al., 2024; Zhou et al., 2023; Wang J. et al., 2024; Zhu et al., 2022; Zhao et al., 2023; Wang Z. et al., 2021). Both the NSCLC and SCLC subgroups demonstrated statistically significant benefits with Tislelizumab. Furthermore, heterogeneity tests for the differences in effect sizes between these subgroups did not reach statistical significance, findings that align with those reported by Ul et al. (2025). This suggests Tislelizumab may possess broadly applicable short-term efficacy across different pathological types of LC. This observation provides a rationale for extending the use of Tislelizumab to a wider spectrum of LC patients.

While our subgroup analysis demonstrated a comparable magnitude of benefit in short-term efficacy (ORR and DCR) for Tislelizumab across both NSCLC and SCLC, it is critical to acknowledge the fundamental differences in the biology and management of these two major LC subtypes. NSCLC, which arises from epithelial cells (e.g., alveolar cells, bronchial cells) and encompasses several subtypes, primarily adenocarcinoma, squamous cell carcinoma, and large cell carcinoma, is often characterized by a slower proliferation rate and the presence of targetable driver oncogenes (e.g., EGFR, ALK, ROS1) in a significant subset of patients, guiding first-line therapy with specific tyrosine kinase inhibitors (TKIs) (Herrera-Juárez et al., 2023; Meyer et al., 2024; Herbst et al., 2018; Sung et al., 2021). In contrast, SCLC is defined by a high-grade neuroendocrine phenotype, exceptionally rapid growth, and an almost universal association with a heavy smoking history. It lacks these actionable driver mutations and is initially highly sensitive to platinum-etoposide chemotherapy, but is notorious for rapid acquisition of chemoresistance and a high propensity for early metastasis, particularly to the brain (Gazdar et al., 2017; Rudin et al., 2021; George et al., 2015).

Therapeutically, the role of ICIs has evolved differently: in NSCLC, ICIs are used across lines of therapy, both as monotherapy in PD-L1 high expressors and, more commonly, in combination with chemotherapy in the first-line setting regardless of PD-L1 status (Herbst et al., 2020; Paz-Ares et al., 2018). In extensive-stage SCLC, the addition of ICIs (e.g., atezolizumab, durvalumab) to first-line platinum-etoposide chemotherapy has become a standard of care, demonstrating a modest but significant improvement in overall survival, albeit the absolute benefits are generally more constrained than those seen in subsets of NSCLC (Horn et al., 2018; Paz-Ares et al., 2019). Therefore, the similar pooled ORR and DCR observed in our analysis for Tislelizumab in both subtypes are particularly noteworthy. They suggest that its mechanism of action—potently blocking the PD-1 pathway to reinvigorate T-cell immunity—is effective against the disparate tumor microenvironments of both NSCLC and SCLC (Cheng Y. et al., 2024; Lu et al., 2024; Zhou et al., 2023; Kumagai et al., 2020). This provides a compelling rationale for the broader application of Tislelizumab-based strategies across the histological spectrum of LC, while underscoring the necessity to evaluate its long-term survival impact within the context of these established, subtype-specific treatment frameworks.

Our analysis, which pooled data from different lines of therapy, found that the point estimates for ORR were numerically higher in the SCLC subgroup [OR = 0.69, 95%CI (0.63, 0.75)] compared to the NSCLC subgroup [OR = 0.48, 95%CI (0.26, 0.74)], though the difference was not statistically significant (p = 0.076). This trend could reflect the particularly high responsiveness of SCLC to first-line chemo-immunotherapy combinations (Cheng Y. et al., 2024), whereas the NSCLC data encompass both later-line monotherapy and first-line combination regimens (Lu et al., 2024; Zhou et al., 2023; Wang J. et al., 2024). It is noteworthy that the high tumor mutational burden (TMB) and immunogenic features of SCLC may contribute to higher initial response rates to immunotherapy, particularly when combined with chemotherapy (Rudin et al., 2021; Hellmann et al., 2018). Therefore, while Tislelizumab demonstrates activity in both histological types, its specific clinical role—whether as first-line combination therapy in SCLC and squamous NSCLC, or as later-line monotherapy in non-squamous NSCLC—is necessarily defined by the distinct therapeutic algorithms for each disease. Future studies with larger sample sizes and stratified by line of therapy are needed to further explore potential efficacy differences within these biologically and epidemiologically distinct entities.

### 4.6 Contextualizing findings with existing evidence and indirect comparisons

When contextualizing our findings within the existing landscape of meta-analyses on PD-1/PD-L1 inhibitors, both similarities and important distinctions emerge. Our results, demonstrating significant improvements in ORR and DCR with Tislelizumab, are consistent with the established efficacy profile of PD-1/PD-L1 blockade in LC, as evidenced by numerous meta-analyses for other agents. For instance, large-scale meta-analyses of pembrolizumab and nivolumab have consistently shown superior ORR and DCR compared to chemotherapy in both first-line and second-line settings for NSCLC, particularly in patients with high PD-L1 expression (Wang et al., 2019b; Reck et al., 2016). Similarly, the lack of a statistically significant improvement in OS and PFS in our RCT analysis, despite a positive trend, echoes the nuanced results seen in some earlier meta-analyses of immunotherapy, where benefits were sometimes confined to specific subgroups or required longer follow-up to become apparent (Chen et al., 2018; Peng and Wu, 2019).

However, a key distinction of our study lies in its specific focus on Tislelizumab. While the efficacy signals (ORR, DCR) appear congruent with the class effect of PD-1 inhibitors, the ITCs cited in our introduction (Guo et al., 2023; Messori et al., 2023) are particularly relevant for cross-sectional comparison. These ITCs, which form a crucial part of the existing evidence base, directly compared Tislelizumab plus chemotherapy with pembrolizumab plus chemotherapy in the first-line advanced NSCLC setting. The findings from Guo et al. (2023) and Messori et al. (2023)—showing no significant differences in PFS, ORR, or grade ≥3 AEs—suggest that Tislelizumab’s efficacy and safety profile may be comparable to that of the established benchmark, pembrolizumab, within the limitations of indirect comparison methodology. Our meta-analysis, by providing pooled estimates specifically for Tislelizumab from both direct and single-arm evidence, complements these ITCs and adds depth to the understanding of this particular agent’s profile.

### 4.7 Safety profile and tolerability in the context of PD-1 inhibitor class effects

Regarding safety, while this meta-analysis did not directly pool AE incidence data due to methodological limitations, reports from the included studies collectively suggest Tislelizumab is generally well-tolerated, exhibiting a safety profile that aligns with the established class effects of PD-1/PD-L1 inhibitors. The spectrum of common AEs predominantly consists of manageable immune-related adverse events (irAEs), such as rash, hypothyroidism, and increased transaminases, alongside chemotherapy-associated toxicities like hematological events (anemia, neutropenia) and gastrointestinal symptoms (nausea, decreased appetite) in combination regimens (Zhou et al., 2023; Wang J. et al., 2024; Zhu et al., 2022; Thompson et al., 2020; Martins et al., 2019).

Crucially, when compared indirectly with other PD-1 inhibitors used in LC, such as pembrolizumab and nivolumab, the safety profile of Tislelizumab appears largely consistent. For instance, the incidence of all-grade TRAEs with Tislelizumab-based regimens in our analysis (ranging from 70% to 95% across studies) is comparable to the 66%–96% range reported for pembrolizumab plus chemotherapy in the KEYNOTE-189 and KEYNOTE-407 trials (Paz-Ares et al., 2018; Gandhi et al., 2018), and to nivolumab-based regimens (Borghaei et al., 2015). Similarly, the spectrum of common irAEs (e.g., thyroid dysfunction, rash, hepatitis) mirrors that well-documented for the drug class (Thompson et al., 2020; Haanen et al., 2017). This cross-trial comparison, while acknowledging inherent limitations, suggests no major, novel safety concerns specific to Tislelizumab.

Beyond incidence rates, the clinical management implications of these AEs are paramount. The irAEs associated with Tislelizumab, akin to other PD-1 inhibitors, are typically manageable with established protocols involving corticosteroids (e.g., prednisone), hormone replacement therapy (e.g., for hypothyroidism), or other immunosuppressants, alongside temporary dose interruption or permanent discontinuation in severe cases (Chen et al., 2018; Haanen et al., 2017; Brahmer et al., 2018). The low incidence of severe (Grade ≥3) specific irAEs in our pooled data (e.g., increased ALT: 0.89%; hypothyroidism: 0.20%; rash: 0.69%) is encouraging and sits within the expected range for this class. For example, the rate of Grade ≥3 pneumonitis with anti-PD-1 agents typically falls below 3% (Naidoo et al., 2016), and our analysis did not identify a significantly higher signal, suggesting a manageable risk profile consistent with its comparators.

However, this analysis represents a limitation requiring careful consideration: we could not directly access and analyze individual patient AE data, relying instead on fragmented descriptions within the published literature to infer safety. The limitations of this approach stem from potential variations across studies in how AEs are defined, graded, and reported, which hinders the derivation of a precise and comparable safety assessment. Although no significant deterioration in OS or PFS was observed in RCTs, and single-arm studies demonstrated favorable efficacy, this does not automatically confirm the treatment’s safety profile as “acceptable” in all contexts. For instance, the occurrence of rare but severe, potentially fatal AEs (e.g., pneumonitis, myocarditis, severe colitis) remains uncertain from our pooled data (Michot et al., 2016; Postow et al., 2018). Moreover, the safety profile in specific populations, such as the elderly or patients with comorbidities, requires further clarification. These aspects necessitate systematic evaluation using standardized, large-sample data.

Notably, the Fc-engineered design of Tislelizumab, which minimizes binding to FcγR on macrophages, is postulated to reduce ADCP (Dahan et al., 2015; Zhang et al., 2018). This theoretical advantage might contribute to a differentiated safety profile by limiting Fc-mediated effector functions that could potentially contribute to certain inflammatory toxicities. While direct comparative safety data from head-to-head trials are lacking to confirm this hypothesis, the pooled safety data from this analysis does not raise any new or unexpected safety signals compared to the established PD-1 inhibitor class, and the incidence of severe irAEs appears numerically comparable to that reported for pembrolizumab and nivolumab (Wang et al., 2019b; Chen et al., 2018; Baxi et al., 2018).

Consequently, conclusions regarding the comprehensive safety of Tislelizumab remain preliminary but reassuring within the context of the known ICI class effects. Its potential risks, especially rare but severe events, should be vigilantly monitored during clinical application, adhering to the same rigorous monitoring, early detection, and prompt management strategies mandated for other ICIs (Chen et al., 2018; Haanen et al., 2017). Future research should prioritize standardized, systematic collection and analysis of safety data from large, real-world cohorts to fully characterize its risk-benefit profile across diverse patient populations and to enable more robust direct or indirect comparisons with other standard immune checkpoint inhibitors.

### 4.8 Conclusion and future directions

In summary, this meta-analysis indicates that Tislelizumab offers significant short-term therapeutic benefits for patients with locally advanced or metastatic LC. However, the current evidence from RCTs is not robust enough to draw definitive conclusions about its long-term survival advantages, potentially due to factors including study design, sample size, and follow-up duration. Regarding safety, preliminary data suggest a potentially manageable profile, although systematic and standardized evaluation data are lacking. Future research should prioritize (Abu Rous et al., 2023): Conducting well-designed, head-to-head RCTs with adequate follow-up to establish the actual differences in OS and PFS between Tislelizumab and standard treatments like chemotherapy (Allemani et al., 2018); Systematically collecting and analyzing Tislelizumab’s safety data to characterize its AE profile, incidence, and management approaches, which is essential for guiding safe clinical application (Frödin, 1996); Investigating the efficacy of Tislelizumab in combination therapies, for instance, alongside chemotherapy, radiotherapy, or other immunotherapies/targeted agents, with the goal of surpassing monotherapy limitations and enhancing patients’ long-term outcomes. Ultimately, only through more comprehensive and in-depth investigation can Tislelizumab’s definitive place in the treatment paradigm for locally advanced or metastatic LC be established.

## 5 Limitations

This study has several limitations that should be considered when interpreting the results. First, the included RCTs and single-arm studies show heterogeneity in baseline patient characteristics, treatment lines, and control group settings, which may impact the estimation of the combined effect size and complicate result interpretation. Second, in the RCT subgroup analyses for OS and PFS, some studies had relatively small sample sizes or insufficient follow-up periods, which may have limited the statistical power of the analyses, especially when evaluating endpoints such as OS, potentially failing to fully capture the true efficacy difference of Tislelizumab. Additionally, the analysis of safety data primarily relies on descriptive information provided by the original studies, lacking standardized and systematic pooled assessments of TRAEs, making comprehensive and accurate depiction of Tislelizumab’s long-term safety profile and the spectrum of rare AEs challenging.

## 6 Conclusion

The results of this meta-analysis indicate that Tislelizumab demonstrates clinically meaningful short-term efficacy in treating patients with locally advanced or metastatic LC. Specifically, Tislelizumab significantly improves patients’ ORR and DCR, with similar treatment effects observed in NSCLC and SCLC subgroups. However, regarding whether it can provide patients with definite long-term survival benefits, i.e., improvements in OS and PFS, the evidence provided by the existing RCTs is insufficient to draw a definitive conclusion. Some studies did not observe statistically significant differences, which may be influenced by factors such as study design, sample size limitations, insufficient follow-up periods, or baseline heterogeneity among patients, but this does not represent a final negation of its long-term potential. Preliminary safety assessments indicate that Tislelizumab is generally well-tolerated, but a systematic safety analysis based on pooled data is lacking. In summary, Tislelizumab offers a promising short-term treatment strategy for patients with locally advanced or metastatic LC. However, its exact long-term clinical value and comprehensive safety profile still depend on future more rigorous, adequately followed-up high-quality randomized controlled trials and standardized safety studies to clarify, thereby providing a more solid evidence base for clinical practice and ultimately establishing Tislelizumab’s position in this treatment field.

## Supplementary material

SUPPLEMENTARY FIGURE S1
**(A)** Sensitivity analysis of overall response rate (ORR) in RCTs; **(B)** Sensitivity analysis of progression-free survival (PFS) in RCTs.

SUPPLEMENTARY FIGURE S2 Sensitivity analysis of overall response rate (ORR) in single-arm studies.

## Glossary

LC: Lung cancer
RCTs: Randomized controlled trials
OR: Odds ratios
CI: Confidence intervals
ORR: Objective response rate
DCR: Disease control rate
OS: Overall survival
PFS: Progression-free survival
NSCLC: Non-Small Cell Lung Cancer
SCLC: Small Cell Lung Cancer
ICIs: Immune checkpoint inhibitors
PD-1: Programmed death receptor 1
PD-L1/2: Programmed death ligand 1/2
FcRs: Fc receptors
ADCP: Antibody-dependent cell-mediated phagocytosis
ITCs: Indirect treatment comparisons
HR: Hazard Ratio
RR: Risk Ratio
AE: Adverse event
NPC: Nasopharyngeal, nasopharyngeal cancer
CR: Complete response
PR: Partial response
SD: Stable disease
TRAEs: Treatment-related adverse events
RoB2: Randomized trials 2.0
MINORS: Methodological Index for Non-Randomized Studies
ES: Effect sizes
ALT: Alanine Aminotransferase
AST: Aspartate Aminotransferase
FcγR: Fcγ receptors
EGFR: Epidermal Growth Factor Receptor
ALK: Anaplastic Lymphoma Kinase
ROS1: ROS Proto-Oncogene 1, Receptor Tyrosine Kinase
TKIs: Tyrosine kinase inhibitors
TMB: Tumor mutational burden
irAEs: Immune-related adverse events
