# Supplementary material for: The efficacy and safety of tislelizumab in the treatment of locally advanced or metastatic lung cancer: a systematic review and meta-analysis
Source: Front Pharmacol. 2025 Oct 28;16:1671018. doi: 10.3389/fphar.2025.1671018 (PMC12602520; doi:10.3389/fphar.2025.1671018)
Supplement: Supplementary file 1 [file Table1.docx]

**Table S1. PubMed Retrieval strategy.**

| No | Query |
| --- | --- |
| #1 | "Lung Neoplasms"[Mesh] |
| #2 | ((((((((((((((((Neoplasms, Pulmonary[Title/Abstract]) OR (Neoplasm, Pulmonary[Title/Abstract])) OR (Pulmonary Neoplasm[Title/Abstract])) OR (Pulmonary Neoplasms[Title/Abstract])) OR (Neoplasms, Lung[Title/Abstract])) OR (Lung Neoplasm[Title/Abstract])) OR (Neoplasm, Lung[Title/Abstract])) OR (Lung Cancer[Title/Abstract])) OR (Cancer, Lung[Title/Abstract])) OR (Cancers, Lung[Title/Abstract])) OR (Lung Cancers[Title/Abstract])) OR (Cancer of Lung[Title/Abstract])) OR (Pulmonary Cancer[Title/Abstract])) OR (Cancer, Pulmonary[Title/Abstract])) OR (Cancers, Pulmonary[Title/Abstract])) OR (Pulmonary Cancers[Title/Abstract])) OR (Cancer of the Lung[Title/Abstract]) |
| #3 | #1 OR #2 |
| #4 | "tislelizumab" [Supplementary Concept] |
| #5 | (((tislelizumab-jsgr[Title/Abstract]) OR (BGB-A317[Title/Abstract])) OR (JHL-2108[Title/Abstract])) OR (tevimbra[Title/Abstract]) |
| #6 | #4 OR #5 |
| #7 | #3 AND #6 |

**Table S2.** Embase Retrieval strategy.

| No | Query |
| --- | --- |
| #28 | #24 AND #27 |
| #27 | #25 OR #26 |
| #26 | tislelizumab:ab,ti OR 'bgb a317':ab,ti OR bgba317:ab,ti OR 'bgn 1':ab,ti OR bgn1:ab,ti OR 'jhl 2108':ab,ti OR jhl2108:ab,ti OR tevimbra:ab,ti OR tirelizumab:ab,ti OR tizveni:ab,ti OR 'vdt 482':ab,ti OR vdt482:ab,ti |
| #25 | 'tislelizumab'/exp |
| #24 | #1 OR #2 OR #3 OR #4 OR #5 OR #6 OR #7 OR #8 OR #9 OR #10 OR #11 OR #12 OR #13 OR #14 OR #15 OR #16 OR #17 OR #18 OR #19 OR #20 OR #21 OR #22 OR #23 |
| #23 | 'tumour, lung':ab,ti |
| #22 | 'tumorigenesis in the lung':ab,ti |
| #21 | 'tumor, lung':ab,ti |
| #20 | 'tumor of the lung':ab,ti |
| #19 | 'pulmonary tumour':ab,ti |
| #18 | 'pulmonary tumorigenesis':ab,ti |
| #17 | 'pulmonary tumor':ab,ti |
| #16 | 'pulmonary neoplasm':ab,ti |
| #15 | 'pulmonary neoplasia':ab,ti |
| #14 | 'neoplastic lung':ab,ti |
| #13 | 'neoplasia of the lung':ab,ti |
| #12 | 'lung tumour':ab,ti |
| #11 | 'lung tumorigenesis':ab,ti |
| #10 | 'lung neoplasms':ab,ti |
| #9 | 'lung neoplasm':ab,ti |
| #8 | 'lung neoplasia':ab,ti |
| #7 | 'bronchopulmonary tumor':ab,ti |
| #6 | 'bronchopulmonary neoplasm':ab,ti |
| #5 | 'bronchopulmonary neoplasia':ab,ti |
| #4 | 'broncho-pulmonary tumor':ab,ti |
| #3 | 'broncho-pulmonary neoplasm':ab,ti |
| #2 | 'lung tumor':ab,ti |
| #1 | 'lung tumor'/exp |

**Table S3.** Methodological Index for Non-Randomized Studies (MINORS)for Single arm studies.

| Methodological Index for Non-Randomized Studies (MINORS)for Single arm studies | | | | | | | | | |
| --- | --- | --- | --- | --- | --- | --- | --- | --- | --- |
| Item  Study | A clearly stated | Inclusion of consecutive  patients | Prospective collection of data | Endpoints appropriate  to the aim of the stud! | Unbiased assessment  of the study endpoint | Follow-up period  appropriate to the aimof the study | Loss to follow up less  than 5% | Prospective  calculation of the  study size | Total score |
| WY Zhu | 2 | 2 | 2 | 2 | 0 | 2 | 2 | 1 | 13 |
| H Zhong | 2 | 2 | 2 | 2 | 0 | 2 | 2 | 1 | 13 |
| ZJ Wang | 2 | 2 | 2 | 2 | 0 | 1 | 2 | 2 | 14 |

Notes: Each item is scored on a 0–2 scale (0 = not reported; 1 = reported but insufficient; 2 = fully reported and methodologically appropriate), with a total score ranging from 0 to 16. Quality grading: 0–12 points: low quality; 13–18 points: moderate quality; 19–24 points: high quality. Literature screening: exclude low-quality studies (e.g., MINORS score [Minimum Information of Report for Non-Comparative Studies] < 12).

**Table S4.** Publish biased assessments.

| Outcome | P value for Begg test | P value for Egger test |
| --- | --- | --- |
| Single-armed experiment | | |
| ORR | 0.602 | 0.873 |
| DCR | 0.602 | 0.209 |
| Randomized controlled trial | | |
| ORR | 0.497 | 0.122 |
| OS | 0.602 | 0.309 |
| PFS | 0.497 | 0.079 |
| DCR | 0.497 | 0.806 |

Notes: Abbreviation: OS: Overall survival; PFS: progression free survival; ORR: Objective response rate.
